# Exploring the influence of steric, electronic and lipophilic descriptors of 1,3-diarly propenones on their anti-inflammatory activity

**Published:** 2010

**Authors:** N.M. Bhatia, K.R. Mahadik, M.S. Bhatia

**Affiliations:** 1 Department of Pharmaceutical Chemistry, Bharati Vidyapeeth College of Pharmacy, Kolhapur; 2 Department of Pharmaceutical Chemistry, BVU Poona College of Pharmacy, Pune, India

**Keywords:** Diarylpropenone, Anti-inflammatory, COX-2, QSAR

## Abstract

**Background and the purpose of the study:**

Various compounds from natural and synthetic origins containing the 1,3-diarylpropenone structure have been reported to produce a variety of biological activities like anti-microbial, anti-inflammatory, vascular muscle relaxant, etc. A systematic analysis of the structural features responsible for anti-inflammatory activity and a possible mode of their actions were proposed to be evaluated by synthesizing a set of compounds, screening them for anti-inflammatory activity and developing a QSAR model.

**Methods:**

Two types of 1,3-diarylpropenone derivatives were synthesized employing the Claisen-Schmidt condensation. These compounds were then screened for their in vivo anti- inflammatory activity by the carrageenin induced rat paw edema method and also for in vitro cyclooxygenase-2 (COX-2) inhibition activity using a colorimetric kit for COX (ovine) inhibitor screening assay. These derivatives and their anti-inflammatory activity data were employed for QSAR analysis on Vlife MDS 3.5 software. The molecules were divided into training and test sets based on observed activity and QSAR models were generated for the training set and validated. The activity of the molecules of the test set was predicted according to the QSAR equation fit. Possible correlation between observed anti-inflammatory activity and in vitro cyclooxygenase-2 inhibition was also studied.

**Results and conclusion:**

Insignificant difference between the observed and predicted biological activity revealed that the selected electronic, steric and lipophilic parameters have a significant correlation (r^2^=0.85) with anti-inflammatory activity of the selected class of compounds. On the basis of results it may be suggested that the 1,3-diaryl-2-propen-1-ones framework is an attractive template for structural optimization to achieve better potency of anti-inflammatory activity. Similarly, the relatively low correlation between anti-inflammatory activity and cyclooxygenase-2 inhibition indicates that other modes of actions may also be responsible for the anti-inflammatory activity of the tested compounds.

## INTRODUCTION

It is well-documented that non-steroidal anti- inflammatory drugs (NSAIDs) exert their effects through inhibition of prostaglandin (PG) synthesis, by blocking cyclooxygenase (COX) activity (1). Two isoforms of the COX enzyme, COX-1 and COX-2, with their functional roles in the maintenance of normal homeostasis and induction of inflammation at inflammatory sites, respectively, were identified early in the past decade (2, 3). Extensive efforts have been made to establish a correlation between structural parameters and biological activity on diverse series of compounds using three-dimensional quantitative structure-activity relationship (3D- QSAR) studies (4).

The presence of reactive a,-unsaturated ketone group in propenones is reported to be responsible for their antimicrobial (5–10), vascular smooth relaxant (11), nitric oxide scavenging (12), cholinesterase inhibitory (13), analgesic and anti-inflammatory (14–16) activities. Progesterone has a a,-unsaturated ketone group and is reported to be a possible contributor to gingival inflammation by upregulation of COX-2 expression and subsequent prostaglandin formation (17). The possibility of such structural similarities contributing to affinity of interaction with common targets could always exist. Here we report the synthesis, anti-inflammatory activity screening and QSAR studies of substituted 1,3-diarylpropenone derivatives. All the compounds were screened for in vivo and in vitro COX-2 inhibitory activity. QSAR analysis was performed to identify the structural features responsible for the targeted activity. The outcome of this work is likely to characterize the structural requirements of 1,3-diaryl-2-propen-1- ones for anti-inflammatory activity, mediated by COX-2 inhibition differentially.

## EXPERIMENTAL

**Figure 1:**
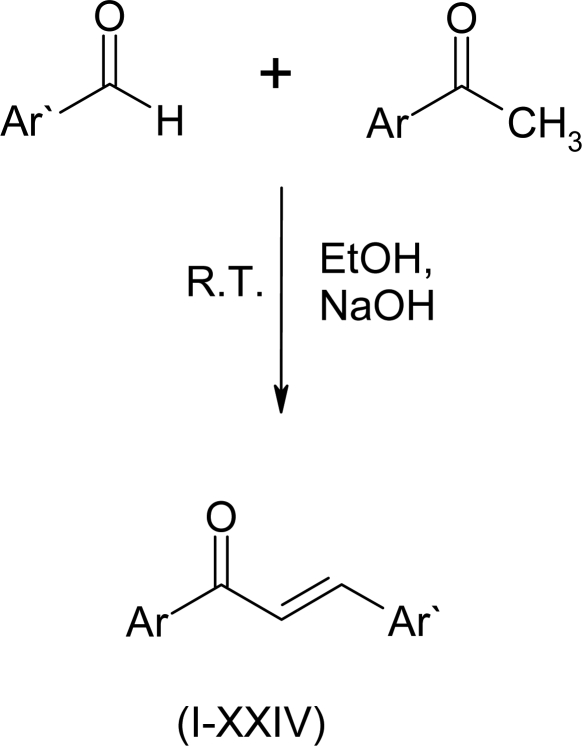
Synthesis of 1,3-diarylprop-2-en-1-ones.

Twenty four 1,3-diarylprop-2-en-1-ones derivatives (Figure. 1) were synthesized by Claisen-Schmidt condensation (18, 19) and evaluated for their anti- inflammatory activity. Among them, chalcones XIII-XVIII are new compounds. The purity of the compound was confirmed by TLC using, silica gel G as stationary phase and ethyl acetate: chloroform (1:2) as mobile phase. The IR spectra were recorded on Jasco FT/IR 4100 spectrophotometer. ^1^H NMR spectra were recorded on Varian NMR 400 MHz spectrometer using DMSO as a solvent and TMS as internal standard. The mass spectra of the compounds were obtained on Micro Mass Q-Top, YA-105. The substituent are shown in table 1.

**Table 1:** Ar and Ar‘ substituents and NMR data of the propenones I–XXIV.

Comp. No.	Ar	Ar'	NMR (DMSO) δ
I	4-methoxyphenyl	4-chlorophenyl	7.65–7.80 (m, 8H, aromatic) 7.50 (d, 1H, CH=CH, *J=*15.7 Hz), 7.53 (d, 1H, CH=CH, *J=*15.7 Hz), 3.84 (s, 3H, OCH3)
II	4-chlorophenyl	4-chlorophenyl	7.21–7.79 (m, 8H, aromatic) 7.49 (d, 1H, CH=CH, *J=*15.6 Hz), 7.22 (d, 1H, CH=CH, *J=*15.6 Hz)
III	4-methyl phenyl	4-chlorophenyl	7.00–7.92 (m, 8H, aromatic) 7.61 (d, 1H, CH=CH, *J=*15.7 Hz), 7.09 (d, 1H, CH=CH, *J=*15.7 Hz), 2.38 (s, 3H, CH3)
IV	4-bromo phenyl	4-chlorophenyl	7.31–7.92 (m, 8H, aromatic) 7.48 (d, 1H, CH=CH, *J=*15.5 Hz), 7.30 (d, 1H, CH=CH, *J=*15.5 Hz)
V	4-nitro phenyl	4-chlorophenyl	7.34–7.76 (m, 8H, aromatic) 7.62 (d, 1H, CH=CH, *J=*15.7 Hz), 7.29 (d, 1H, CH=CH, *J=*15.7 Hz)
VI	NN-dimethyl-4-aminophenyl	4-chlorophenyl	7.49–8.10 (m, 8H, aromatic) 7.52 (d, 1H, CH=CH, *J=*15.8 Hz), 7.22 (d, 1H, CH=CH, *J=*15.8 Hz), 2.24 (s, 6H, (N(CH_3_)_2_)
VII	4-methoxyphenyl	3,4,5-trimethoxy phenyl	7.35–7.71 (m, 6H, aromatic) 7.42 (d, 1H, CH=CH, *J=*15.6 Hz), 7.14 (d, 1H, CH=CH, *J=*15.6 Hz), 3.88 (s, 12H, OCH3)
VIII	4-chlorophenyl	3,4,5-trimethoxy phenyl	7.18–7.79 (m, 6H, aromatic) 7.58 (d, 1H, CH=CH, *J=*15.7 Hz), 7.21 (d, 1H, CH=CH, *J=*15.7 Hz). 3.84 (s, 9H, OCH3)
IX	4-methyl phenyl	3,4,5-trimethoxy phenyl	7.00–7.92 (m, 6H, aromatic) 7.51 (d, 1H, CH=CH, *J=*15.5 Hz), 7.09 (d, 1H, CH=CH, *J=*15.5 Hz), 3.84 (s, 9H, OCH3), 2.34 (s, 3H, CH3)
X	4-bromo phenyl	3,4,5-trimethoxy phenyl	7.11–7.52 (m, 6H, aromatic) 7.58 (d, 1H, CH=CH, *J=*15.7 Hz), 7.29 (d, 1H, CH=CH, *J=*15.7 Hz) 3.88 (s, 9H, OCH3)
XI	4-nitro phenyl	3,4,5-trimethoxy phenyl	7.14–7.46 (m, 6H, aromatic) 7.60 (d, 1H, CH=CH, *J=*15.8 Hz), 7.22 (d, 1H, CH=CH, *J=*15.8), 3.72 (s, 9H, OCH3)
XII	NN-dimethyl-4-aminophenyl	3,4,5-trimethoxy phenyl	7.39–7.70 (m, 6H, aromatic) 7.68 (d, 1H, CH=CH), 7.26 (d, 1H, CH=CH), 3.80 (s, 9H, OCH3), 2.31 (s, 6H, (N(CH_3_)_2_)
XIII	4-methoxyphenyl	Indol-3-yl	7.45–8.12 (m, 8H, aromatic) 7.38 (s, 1H, NH), 6.71 (s, 1H, 2-indole), 7.68 (d, 1H, CH=CH), 7.22 (d, 1H, CH=CH), 3.84 (s, 3H, OCH3) and J(Vinylic H)=15.6 Hz.
XIV	4-chlorophenyl	Indol-3-yl	7.51–7.79 (m, 8H, aromatic), 7.41 (s, 1H, NH), 6.71 (s, 1H, 2-indole), 7.62 (d, 1H, CH=CH, *J=*15.7 Hz), 7.27 (d, 1H, CH=CH, *J=*15.7 Hz)
XV	4-methyl phenyl	Indol-3-yl	7.40–7.92 (m, 8H, aromatic), 7.34 (s, 1H, NH), 6.61 (s, 1H, 2-indole), 7.57 (d, 1H, CH=CH, *J=*15.6 Hz), 7.29 (d, 1H, CH=CH, *J=*15.6 Hz), 2.29 (s, 3H, CH3)
XVI	4-bromo phenyl	Indol-3-yl	7.45–7.88 (m, 8H, aromatic), 7.39 (s, 1H, NH), 6.71 (s, 1H, 2-indole), 7.61 (d, 1H, CH=CH, *J=*15.8 Hz), 7.22 (d, 1H, CH=CH, *J=*15.8 Hz)
XVII	4-nitro phenyl	Indol-3-yl	7.51–7.91 (m, 8H, aromatic), 7.42 (s, 1H, NH), 6.77 (s, 1H, 2-indole), 7.54 (d, 1H, CH=CH, *J=*15.9 Hz), 7.14 (d, 1H, CH=CH, *J=*15.9)
XVIII	N,N-dimethyl-4-aminophenyl	Indol-3-yl	7.49–8.10 (m, 8H, aromatic) 7.34 (s, 1H, NH), 6.66 (s, 1H, 2-indole), 7.50 (d, 1H, CH=CH, *J=*15.5 Hz), 7.20 (d, 1H, CH=CH, *J=*15.5 Hz), 2.29 (s, 6H, (N(CH_3_)_2_)
XIX	4-methoxyphenyl	N,N-dimethyl-4 aminophenyl	7.45–7.70 (m, 8H, aromatic) 7.58 (d, 1H, CH=CH, *J=*15.5 Hz), 7.21 (d, 1H, CH=CH, *J=*15.5 Hz), 3.82 (s, 3H, OCH3). 2.27 (s, 6H, (N(CH_3_)_2_)
XX	4-chlorophenyl	N,N-dimethyl-4 aminophenyl	7.61-7.99 (m, 8H, aromatic) 7.62 (d, 1H, CH=CH, *J=*15.6 Hz), 7.28 (d, 1H, CH=CH, *J=*15.6 Hz). 2.31 (s, 6H, (N(CH_3_)_2_)
XXI	4-methyl phenyl	N,N-dimethyl-4 aminophenyl	7.20–7.72 (m, 8H, aromatic) 7.46 (d, 1H, CH=CH, *J*=15.5 Hz), 7.19 (d, 1H, CH=CH, *J=*15.5 Hz), 2.33 (s, 3H, CH3). 2.34 (s, 6H, (N(CH_3_)_2_)
XXII	4-bromo phenyl	N,N-dimethyl-4 aminophenyl	7.42–7.89 (m, 8H, aromatic) 7.59 (d, 1H, CH=CH, *J=*15.7 Hz), 7.29 (d, 1H, CH=CH, *J=*15.7 Hz). 2.24 (s, 6H, (N(CH_3_)_2_)
XXIII	4-nitro phenyl	N,N-dimethyl-4 aminophenyl	7.27-7.86 (m, 8H, aromatic) 7.54 (d, 1H, CH=CH, *J=*15.8 Hz), 7.22 (d, 1H, CH=CH, *J=*15.8 Hz). 2.22 (s, 6H, (N(CH_3_)_2_)
XXIV	N,N-dimethyl-4 aminophenyl	N,N-dimethyl-4 aminophenyl	7.46–8.05 (m, 8H, aromatic) 7.57 (d, 1H, CH=CH, *J=*15.5 Hz), 7.30 (d, 1H, CH=CH, *J=*15.5 Hz), 2.24 (s, 9H, 2(N(CH_3_)_2_)

### 

#### Procedure for synthesis of 1,3-diarylprop-2-en-1-ones (I-XXVI)18

To a stirring solution of aldehyde (0.01 mol) in ethanol at room temperature was added 10 ml of 10% sodium hydroxide. To this mixture was added 0.01 mol of the corresponding ketone dropwise with constant stirring. The stirring was continued until final product was precipitated. The product was filtered, washed with water and recrystallized from ethanol to give the respective 1,3-diarylprop-2-en-1-ones. The NMR data of synthesized 1,3-disubstituted prop-2-enones are shown in table 1.

#### Anti-inflammatory activity

##### In vivo anti-inflammatory activity

The anti-inflammatory activity was evaluated against carrageenin induced paw edema in albino rats of either sex weighing 150–250g each (20, 21). Prior to experiments 0.05ml of freshly prepared suspension of carrageenin (1.0%) in 0.9% saline was injected. One group of six rats was kept as control and other groups were pre-treated with the test and standard drugs at doses plethysmographically of 100 mg/ kg orally. Rat paw volume was measured before and 3 hrs after treatment with carrageenin. Increase in volume of paw in each group was measured and percentage of anti-inflammatory activity was calculated by formula:

$$\text{Percentage anti}-\text{inflammatory avtivity}=\left[\text{1}-\frac{{\text{V}}_{\text{t}}}{{\text{V}}_{\text{c}}}\right]\times \text{100}$$

Where V_t_ and V_c_ are the volume of paw in edema in drug treated and control group respectively. The ED_80_ values calculated from the data generated are shown in table 2, where ED_80_ is the concentration of the drug to produce 80% response in the animal (21, 22).

**Table 2:** Anti-inflammatory activity of synthesized compounds I-XXIV.

Comp. No	% Reduction in Edema (±SD)*	pED80
I	46.11 (±1.49)	−0.04844
II	97.24 (±0.92)	0.2757
III	89.22 (±1.06)	0.2382
IV	94.60 (±0.74)	0.2636
V	78.44 (±0.67)	0.1823
VI	34.05 (±0.84)	−0.1801
VII	60.20 (±2.16)	0.0673
VIII	88.44 (±0.97)	0.2344
IX	26.65 (±0.47)	−0.2865
X	85.32 (±1.46)	0.2188
XI	33.06 (±0.84)	−0.1928
XII	16.54 (±1.24)	−0.4937
XIII	56.89 (±0.92)	0.0427
XIV	97.08 (±1.40)	0.2749
XV	75.94 (±1.12)	0.1682
XVI	93.09 (±0.53)	0.2566
XVII	83.24 (±0.45)	0.2081
XVIII	52.92 (±0.96)	0.0113
XIX	69.88 (±1.07)	0.1321
XX	62.15 (±0.81)	0.0811
XXI	42.18 (±0.58)	−0.0870
XXII	60.58 (±1.30)	0.07007
XXIII	27.62 (±1.35)	−0.2710
XXIV	12.82 (±1.04)	−0.6043
Ibuprofen	88.09 (±0.86)	

##### In vitro testing [Colorimetric COX (ovine) Inhibitor Screening Assay Kit]

One hundred and sixty microliters of the assay buffer and 10 µl of heme were added to the background wells. Similarly 150 µl of the assay buffer, 10 µl of heme and 10 µl of enzyme (COX-2) was added to each of the 3 (100% Initial Activity Wells) wells as well as to 3 (Inhibitory Activity Wells) wells. Then 10 µl of inhibitor was added to the inhibitory activity wells while 10 µl of the solvent in which the inhibitors were dissolved was added to each of the 100% initial activity and background wells. These plate were carefully shaken for a few seconds and incubated for 5 minutes at 25°C and then 20 µl of the colorimetric substrate solution was added to all wells. Then 20 µl of arachidonic acid was added to all wells and the plates were shaken carefully for a few seconds and incubated at 25°C for 5 min. The absorbance was measured at 590 nm using plate reader. The absorbance and the calculated IC_80_ values are shown in table 3 where IC_80_ is the concentration of drug required to inhibit 80% of the enzyme.

**Table 3:** Percentage (%) of COX-2 inhibitory activity of the synthesized compounds I-XXIV.

Comp. No	Conc. µg/ml	Average Absorbance (600 nm)
Background Well	-	0.248
100% Initial Activity Well	-	0.420
I	10	0.272
II	10	0.367
III	10	0.359
IV	10	0.312
V	10	0.328
VI	10	0.258
VII	10	0.306
VIII	10	0.366
IX	10	0.266
X	10	0.351
XI	10	0.260
XII	10	0.297
XIII	10	0.317
XIV	10	0.398
XV	10	0.316
XVI	10	0.305
XVII	10	0.358
XVIII	10	0.330
XIX	10	0.335
XX	10	0.324
XXI	10	0.297
XXII	10	0.317
XXIII	10	0.275
XXIV	10	0.262

#### QSAR Analysis

##### Data Set

The builder module of the Vlife MDS was used to generate molecular models of a series of chalcone derivatives. These were then energy-minimized using the Merck Molecular Force Field (MMFF). The structures were energy-minimized until the root mean square (rms) gradient reached to the value of 0.001 kcal mo1^−1^A°. The charge equilibration method was used to assign atomic partial charges to each of the compounds. pED80 values that is the negative logarithms to the base 10 of the IC_80_ values, for each compound were used for the present QSAR study. The physicochemical properties of each compound were specified using various descriptors, which delineate liphophillic, conformational, electronic, spatial, structural, thermodynamic and quantum mechanical information. The selected descriptors included the X-component of dipole moment (XD), valence molecular connectivity index (chiV3) and log of partition coefficient calculated as per a atomic contribution model (SLogP). Based on appropriate representation of quantitative ub-classes of biological activity, from the 24 compounds 18 were randomly chosen for the training set and the remaining compounds were used as test set for checking the external predictivity of generated QSAR models. The selected model developed by using the training set was used to predict the activity of the compounds in the test set and subsequently to predict the activity of all 24 compounds by leaving a group out every time.

##### Full Search Multiple Linear Regression Method

A relationship between independent and dependent variables (physicochemical descriptors and biological activities, respectively) were determined statistically using regression analysis. Linear regression was achieved by fitting a best-fit straight line to the data using the least squares method. Descriptors that are included in a reasonable QSAR equation should exhibit high correlation to biological activity and low inter-correlation so that a diverse set of independent variables with high correlation to activity are selected. High correlation with targeted activity followed by low inter-correlation between descriptors were the criteria which were used for selection of descriptors for equation and the quality of fit for a regression equation was assessed relative to its correlation coefficient and standard deviation. The F value represents the level of statistical significance of the regression. Quality of selected models was further ascertained to select the best model from cross-validated squared correlation coefficient (q^2^). In the relation for q^2^ shown below,

$$q^{2}=1-\text{PRESS}/\text{TOTAL}$$

Σ(Y_predicted_ -Y_observed_)^2^ is the predictive error sum of squares (PRESS). S(Y_obser ved_ -Y_mean_)^2^ is the total sum of squares (TOTAL), where Y_predicted_, Y_observed_, and Y_mean_ are the predicted, observed, and mean values of activity, respectively. For a regression model, r^2^ was used to describe the fitness of data and fitness is considered to improve as r^2^ approaches 1. Thus models having correlation coefficient above 0.7 were used to check the external predictivity while the significance of the model was decided on the basis of F value. Models showing q^2^ below 0.6 were discarded. The selected model fulfilling above criteria is given in table 4 and correlation matrix of the descriptors in the selected model is given is table 5.

**Table 4:** QSAR model generated for the in vivo anti-inflammatory activity.

QSAR model	N	r^2^	q^2^	F value	Pred r^2^	SE
pED80=0.3952 SlogP-0.0214 chiV3-0.053 XD-1.0123	19	0.794	0.657	31.00	0.718	0.105

**Table 5:** Correlation matrix of descriptors used in the study.

	Slog P	chiV3	XD
Slog P	1.000000	0.036276	0.214725
chiV3	0.036276	1.000000	0.090551
XD	0.214725	0.090551	1.000000

*All the values are calculated on the Vlife sciences MDS 3.5.

##### Activity prediction

To assess a QSAR model systematically, a reliable validation is required. Usually, a QSAR model is evaluated by the predictive results for the given data-set. Selected models having r^2^ above 0.7 were checked for their external predictivity. The observed and the predicted values for anti-inflammatory activity are shown in table 6.

**Table 6:** Observed in vivo anti-inflammatory activity, predicted activity and residuals.

Comp. No.	Observed pED80	Predicted pED80*	Residuals*
I	−0.048	−0.076	0.028
II	0.275	0.251	0.025
III	0.238	0.223	0.015
IV	0.263	0.301	−0.037
V	0.182	0.112	0.070
VI	−0.180	−0.020	−0.160
VII	0.067	−0.023	0.090
VIII	0.234	0.096	0.138
IX	−0.286	−0.067	−0.219
X	0.218	0.153	0.066
XI	−0.192	−0.079	−0.114
XII	−0.493	−0.358	−0.135
XIII	0.042	0.095	−0.052
XIV	0.274	0.267	0.008
XV	0.168	0.088	0.080
XVI	0.256	0.349	−0.092
XVII	0.208	0.173	0.035
XVIII	0.011	−0.146	0.157
XIX	0.132	−0.160	0.292
XX	0.081	0.064	0.017
XXI	−0.087	−0.249	0.162
XXII	0.070	0.100	−0.030
XXIII	−0.271	−0.206	−0.065
XXIV	−0.604	−0.460	−0.144

* Calculated by Vlife MDS 3.5

## RESULTS AND DISCUSSION

In the present work some 1,3-diarylpropenone derivatives were synthesized by nucleophilic condensation of aldehyde and ketones as per reported procedure. The purity and structure products were confirmed by chromatographic and spectral data analysis. The IR absorption bands in the range from 1540–1559 and 1655–1686 cm^−1^ indicated the presence of a conjugated carbonyl group (C=C-C=O) and confirmed the formation of the propenone derivatives. The ^1^H NMR spectrum of these compounds shows the presence of two doublets of vinylic protons at δ 7.09 to 7.68 (*J=*15.5 to 15.9 Hz) indicating presence of only the E-isomers of all compounds including the compounds with the Ar‘, Indol-3-yl substituent.

**Figure 2:**
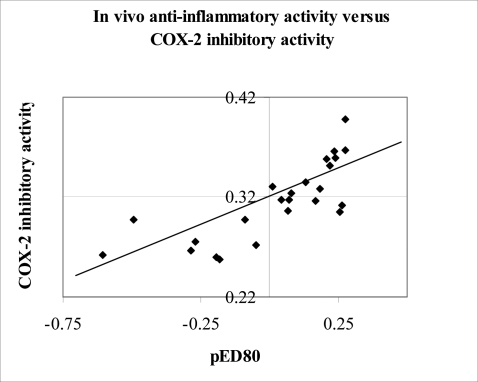
In vivo anti-inflammatory activity versus COX-2 inhibitory activity.

The compounds were screened for anti-inflammatory activity by carrageenin induced rat paw edema method, using ibuprofen as standard. The COX-2 inhibitory activity was also checked and a comparative account of both activities for the 24 synthesized compounds is presented graphically in figure 2. The correlation coefficient for in vitro COX-2 inhibition versus the in vivo anti-inflammatory activity is 0.61. This value is an indication that COX-2 inhibition may not be the sole mechanism by which these compounds act as anti-inflammatory agents and other mechanisms like inhibition of the lipoxygenase and heme oxygenase-1 by the active compounds should also be studied.

Similarly, amongst the compounds showing good anti-inflammatory activity, the low residuals of compound numbers II, III, IV and XIV indicate good predictability of the QSAR model for these compounds while in the case of the remaining active compounds, V, VIII, X, XVI and XVII, the predicted activity deviates highly from the observed activity. These results also indicate that the selected set of descriptors have relevance to a certain target receptor whereas some other modes of action involving different receptors may have a significant contribution in the observed anti-inflammatory action of the compounds whose predicted activities show large deviations from their observed activities.

The selected QSAR model was found best to express anti-inflammatory activity as confirmed by validation of the model judging internal and external predictivity and other statistical terms like the predicted r^2^, F value and q^2^. The variable terms in the equation together yield a good correlation to the targeted activity showing low correlation among themselves and hence show a diverse set of variables influencing biological activity. As indicated in table 5 the anti-inflammatory activity increases by increase in SlogP and decreases in chiV3 and XD within the limits explored for this work.

As it is evident from the compounds producing better activity, an electronegative atom like chlorine or bromine are distant from the electron rich pyrrole of the indolyl substituent and structural features enhancing the dipole are likely to improve activity. Similarly the increase in the multiplicity of substituents could positively contribute to chiV3 and hence compounds with multiple substituents like methoxy and dimethylamino are less active. Atomic contributions to partition coefficient which would increase hydrophilicity and polarity and hence decrease in the partition coefficient within the range covered by the compounds of this work could possibly improve anti-inflammatory activity. The presence of a 4-bromophenyl and indolyl substituents in compound XIV resulted in good anti- inflammatory activity but the COX-2 inhibition was relatively low.

## CONCLUSION

QSAR analysis on a structurally diverse but small set of propenones has led to the identification of structural, electronic and steric factors contributing to anti-inflammatory activity and COX-2 selectivity. Robust statistical parameters handled the physicochemical descriptors effectively to rule out chance co-relations with an acceptable level of approximations. Evaluation and comparison of the QSAR analysis with the results of COX-2 selectivity and potency led to understanding that though, COX-2 inhibition may possibly be the mechanism for the anti-inflammatory action but it does not solely account for the anti- inflammatory activity shown by all the synthesized propenone derivatives. The correlation of the exhibited activity is required to be explored with other targets for anti-inflammatory compounds like heme oxygenase-1, lipoxygenase, etc.

Moreover, the awareness and understanding of the descriptors influencing both the affinity and selectivity properties of these compounds could provide an opportunity to understand and optimize appropriate features of the ligand structures which correlate with the biological data. As a consequence, the outcome of this study could be useful as a guide for further development of safer COX-2 inhibitors and anti-inflammatory drugs.
